# A Systematic Review Exploring the Range of Renal Complications of Human Immunodeficiency Virus

**DOI:** 10.7759/cureus.36755

**Published:** 2023-03-27

**Authors:** Feisal M Yussuf, Ahmed Barbarawi, Mohammed A Nor, Abdirazak I Ali, Ibrahimkhalil M Sheikh, Oboseh J Ogedegbe, Godfrey Tabowei, Abdulmalik Jimoh, Godwin E Ozokolie, Osahon Igbinomwanhia

**Affiliations:** 1 General Practice, Antaliya Hospital, Garissa, KEN; 2 Internal Medicine, University of Minnesota, Minneapolis, USA; 3 Internal Medicine, Norman Bethune Health Science Center of Jilin University, Jilin, CHN; 4 Pediatrics, University of Minnesota, Minnespolis, USA; 5 General Practice, Erciyes University, Kayseri, TUR; 6 Internal Medicine, Lifeway Medical Center, Abuja, NGA; 7 Internal Medicine, California Institute of Behavioral Neurosciences & Psychology, Fairfield, USA; 8 Internal Medicine, Mount Horeb Clinic and Dialysis Center, Warri, NGA; 9 Surgery, Ahmadu Bello University Teaching Hospital, Zaria, NGA; 10 Public Health, Glasgow Caledonian University, Glasgow, GBR; 11 Medicine and Surgery, Igbinedion University Okada, Okada, NGA

**Keywords:** kidney disease, renal complication, cd4 t-cells, hiv-associated immune complex kidney disease (hivick), hiv associated nephropathy (hivan), hiv aids, hiv-1

## Abstract

Human immunodeficiency virus (HIV) is a viral infection which progressively leads to acquired immunodeficiency syndrome (AIDS) in the absence of treatment. This happens through the destruction of crucial cells in the immune system, such as the helper T cells, dendritic cells, and macrophages. Since the first case was isolated in the 20th century, the disease has spread rapidly among humans, with significant renal, cardiovascular, respiratory, and neurological complications. It is predominantly sexually transmitted but non-sexual transmission. A relationship between HIV and renal diseases has been suggested for a long time, but only a few systematic studies have centered on this association. This systematic review aims to analyze the possible association between HIV and renal diseases as well as the range and pathogenesis of these renal diseases. HIV remains a critical infectious disease globally, inciting substantial morbidity and mortality. Studies have shown that people living with HIV (PLWH) are at increased risk of acute and chronic kidney disease. This review is based on Preferred Reporting Items for Systematic Reviews and Meta-Analyses (PRISMA) guidelines. PubMed, Google Scholar, and Cochrane databases were searched exhaustively using the inclusion criteria of free full-text English papers that have exclusively studied humans in the last 20 years. Sixteen articles were selected including a systematic review, observational studies, and comprehensive narrative reviews on the role of HIV in the etiology of renal diseases, and were systemically reviewed and analyzed to elicit the wide range of possible renal complications resulting from HIV infection.

## Introduction and background

Acquired immunodeficiency syndrome (AIDS) is caused by human immunodeficiency virus (HIV). HIV is a lentivirus and belongs to the *Retroviridae* family and HIV isolates are currently grouped into HIV-type 1 (HIV-1) and HIV-type 2 (HIV-2) [1]. It was, however, first acknowledged as a new disease entity in the summer of 1981 in the United States when young homosexual men began to suddenly die from opportunistic infections while developing Kaposi’s sarcoma, which was a rare skin condition at the time [2]. A year later (1982), the same disease entity was diagnosed in Europe, and it was named AIDS by the Center for Disease Control (CDC) [2]. Since the first diagnosed case, about 79.3 million (55.9-110 million) have been infected with the virus [3], while over 25 million people have died already [4]. In addition, about two million new cases are diagnosed annually [4]. Heterosexual viral transmission remains the most common mechanism of spread, responsible for about 85% of HIV-1 infections, and Southern Africa remains the global epicenter of this pandemic [4]. The main worldwide agent of AIDS is HIV-1, while HIV-2 is restricted to some regions of Western and Central Africa [5].

HIV possesses three critical genes, which are vital and indispensable to its replication cycle and, therefore, its pathogenesis. These genes include the group antigen (*gag*), deoxyribonucleic acid polymerase (*pol*), and envelop protein (*env*) [6]. Identical to other retroviruses, the *gag* gene encodes the structural proteins of the core (p24, p7, p6) and matrix (p17), and the *env* gene encodes the viral envelope glycoproteins gp120 and gp41, which are responsible for the recognition of cell surface receptors [7]. The *pol* gene encodes for the enzymes responsible for viral replication, including the reverse transcriptase, which converts viral RNA into DNA, the integrase that incorporates the viral DNA into host chromosomal DNA (the provirus), and the protease that cleaves large Gag and Pol protein precursors into their components [5]. The infective etiopathogenesis of HIV-1 begins when the virus gains entry to cells without causing immediate lethal damage. This access into cells triggers intracellular signal cascades, which then expedites the duplication of viral particles. The two envelope proteins, the external glycoprotein (gp120) and the transmembrane protein (gp41), establish the spikes on the virion’s surface [7]. The external glycoprotein gp120 attaches to the cell membrane to facilitate entry by first binding to the CD4+ receptor [8]. This is followed by interactions between the virus and the chemokine co-receptors, which include C-C motif chemokine receptor 5 (CCR5) and C-X-C chemokine receptor type 4 (CXCR4), thereby triggering irreversible conformational alterations [9]. This fusion event occurs within minutes by pore formation [10], and releases the viral core into the cell cytoplasm.

The next stage of genomic imprinting involves the reverse transcription of the viral genome into DNA by the virus’ reverse transcriptase enzyme [4]. Other essential non-structural viral proteins such as Vpr, Tat, and Nef play crucial roles in the life cycle of HIV. Vpr is responsible for promoting nuclear importation of the viral pre-integration complex, a major phase in integrating viral particles into the host genetic makeup [11]. Tat increases the effectiveness of gene transcription and induces the expression of proinflammatory cytokines [12]. Nef plays crucial roles in T-cell activation, decreasing cell surface CD4 expression, viral transcription amplification, and stimulation of cell signaling pathways [13]. The dissimilarities between HIV-1 and HIV-2 viruses are seen in their genome structure. However, the basic organization, which is the presence of the three foundational genes (*gag, pol*, and *env*), is constant for all retroviruses. In addition to these three foundational genes, the HIV-1 and HIV-2 genomes possess a complex combination of additional regulatory genes [5]. While both viruses can cause AIDS, HIV-2 is less virulent and takes a longer clinical progression course to AIDS. Furthermore, HIV-1 causes central nervous system disease more regularly [11]. HIV entry into renal cells transpires in a CD4+-independent manner, due to the fact that neither podocytes nor renal tubular epithelial cells express CD4+ nor the co-receptors [14]. However, productive infection of renal cells has been shown in the setting of direct cell-cell interaction with both infected CD4+ T cells and macrophages [14].

Several mechanisms may contribute to kidney disease in people living with HIV (PLWH), including direct renal damage resulting from intrarenal HIV infection and gene expression, immune dysregulation, treatment toxicity, comorbidities, and co-infections [14]. Renal diseases have increasingly become a very important cause of morbidity and mortality in PLWH. This systematic review seeks to explore the wide range of renal diseases associated with HIV infection.

## Review

Methods

This systematic review was based on the Preferred Reporting Items for Systematic Reviews and Meta-Analyses (PRISMA) 2020 guidelines [15].

Eligibility Criteria

We identified specific inclusion criteria to select the studies relevant to our systematic review. Studies evaluating the role of HIV in the etiopathogenesis of various renal diseases were selected as the primary target of our research. We selected studies published from 2003 to 2023, including systematic reviews, traditional reviews, case reports, reviews of literature, and observational studies. Articles published in languages other than English were excluded, along with studies unrelated to the topic, and abstracts with no access to full-text articles. In addition, animal studies, conference abstracts, and duplicated articles were also excluded.

Selection Strategy

Two reviewers selected the articles independently using the same search strategy in all three databases. At first, articles were screened from the title of articles and abstracts and then later by reading full-text articles. If contradicting results regarding the article's eligibility occurred, reviewers assessed the full-text article until the group reached a consensus.

Databases and Search Strategy

We searched electronic medical databases PubMed, Google Scholar, and Cochrane from January 2003 to January 2023 to elicit all English human studies assessing the role of HIV in the pathogenesis of renal diseases. Keywords used in all search engines included: 'HIV' or 'human immunodeficiency virus' or 'AIDS' or 'acquired immunodeficiency syndrome' AND 'renal failure' or 'kidney failure' or 'renal impairment' or 'kidney impairment' or 'renal insufficiency' or 'kidney insufficiency' or 'renal disease' or 'kidney disease' or 'acute kidney injury'.

Analysis of Study Quality/Bias

The full articles remaining were assessed for quality assessment and risk of bias using tools depending on the study type: Cohort Studies, Newcastle Ottawa Scale (NOS) [16]; Systematic reviews and Meta-analyses, Assessment of Multiple Systematic Reviews 2 (PRISMA 2020 Checklist) [15]; and Narrative reviews, Scale for the Assessment of Narrative Review Articles (SANRA) [17]. Each assessment tool had its criteria and different scoring, and each selected study was assessed for risk of bias by two reviewers independently using commonly used tools for each type of study, and only studies that scored more significantly than 70% were included in this review. A score of at least 70% for each assessment tool was accepted. Table 1 expatiates the questions used to assess the qualities of the papers included in the study.

**Table 1:** Quality assessment of each study PRISMA: Preferred Reporting Items for Systematic Reviews and Meta-analyses; SANRA: Scale for the assessment of Narrative Review Articles *Maximum of two points are allotted in this category.

Quality Assessment Tool	Type of Study	Items and Their Characteristics	Total Score	Accepted Score (>70)	Accepted Studies
PRISMA [15]	Systematic Review and Meta-analysis	34 Items: 1) Did the review authors Identify the report as a systematic review? 2) Did the review authors see the PRISMA 2020 for Abstracts checklist? 3) Did the review authors describe the rationale for the review in the context of existing knowledge? 4) Did the review authors provide an explicit statement of the objective(s) or question(s) the review addresses? 5) Did the review authors specify the inclusion and exclusion criteria for the review and how studies were grouped for the syntheses? 6) Did the review authors specify all databases, registers, websites, organizations, reference lists, and other sources searched or consulted to identify studies? Specify the date when each source was last searched or consulted. 7) Did the review authors present the full search strategies for all databases, registers, and websites, including any filters and limits used? 8) Did the review authors specify the methods used to decide whether a study met the inclusion criteria of the review, including how many reviewers screened each record and each report retrieved, whether they worked independently, and if applicable, details of automation tools used in the process? 9) Did the review authors specify the methods used to collect data from reports, including how many reviewers collected data from each report, whether they worked independently, any processes for obtaining or confirming data from study investigators, and if applicable, details of automation tools used in the process? 10a) Did the review authors list and define all outcomes for which data were sought? Specify whether all results that were compatible with each outcome domain in each study were sought (e.g., for all measures, time points, analyses), and if not, the methods used to decide which results to collect. 10b) Did the review authors list and define all other variables for which data were sought (e.g., participant and intervention characteristics, funding sources)? Describe any assumptions made about any missing or unclear information. 11) Did the review authors specify the methods used to assess risk of bias in the included studies, including details of the tool(s) used, how many reviewers assessed each study and whether they worked independently, and if applicable, details of automation tools used in the process? 12) Did the review authors specify for each outcome the effect measure(s) (e.g., risk ratio, mean difference) used in the synthesis or presentation of results? 13a) Did the review authors describe the processes used to decide which studies were eligible for each synthesis (e.g., tabulating the study intervention characteristics and comparing against the planned groups for each synthesis? 13b) Did the review authors describe any methods required to prepare the data for presentation or synthesis, such as handling of missing summary statistics, or data conversions? 13c) Did the review authors describe any methods used to tabulate or visually display results of individual studies and syntheses? 13d) Did the review authors describe any methods used to synthesize results and provide a rationale for the choice(s)? If meta-analysis was performed, describe the model(s), method(s) to identify the presence and extent of statistical heterogeneity, and software package(s) used. 13e) Did the review authors describe any methods used to explore possible causes of heterogeneity among study results (e.g., subgroup analysis, meta-regression)? 13f) Did the review authors describe any sensitivity analyses conducted to assess the robustness of the synthesized results? 14) Did the review authors describe any methods used to assess the risk of bias due to missing results in a synthesis (arising from reporting biases)? 15) Did the review authors describe any methods used to assess certainty (or confidence) in the body of evidence for an outcome? 16a) Did the review authors describe the search and selection process results, from the number of records identified in the search to the number of studies included in the review, ideally using a flow diagram? 16b) Did the review authors cite studies that might appear to meet the inclusion criteria but which were excluded, and explain why they were excluded? 17) Did the review authors cite each included study and present its characteristics? 18) Did the review authors present bias risk assessments for each included study? 19) Did the review authors for all outcomes present for each study: (a) summary statistics for each group (where appropriate) and (b) an effect estimate and its precision (e.g., confidence/credible interval), ideally using structured tables or plots? 20a) Did the review authors for each synthesis briefly summarise the characteristics and risk of bias among contributing studies? 20b) Did the review authors present the results of all statistical syntheses conducted? If meta-analysis was done, present for each the summary estimate and its precision (e.g., confidence/credible interval) and measures of statistical heterogeneity. If comparing groups, describe the direction of the effect. 20c) Did the review authors present results of all investigations of possible causes of heterogeneity among study results? 20d) Did the review authors present the results of all sensitivity analyses conducted to assess the robustness of the synthesized results? 21) Did the review authors present assessments of risk of bias due to missing results (arising from reporting biases) for each synthesis assessed? 22) Did the review authors present certainty (or confidence) assessments in the body of evidence for each outcome assessed? 23a) Did the review authors provide a general interpretation of the results in the context of other evidence? 23b) Did the review authors discuss any limitations of the evidence included in the review? 23c) Did the review authors discuss any limitations of the review processes used? 23d) Did the review authors discuss the implications of the results for practice, policy, and future research? 24a) Did the review authors provide registration information for the review, including register name and registration number, or state that the review was not registered? 24b) Did the review authors Indicate where the review protocol can be accessed or state that a protocol was not prepared? 24c) Did the review authors describe and explain any amendments to the information provided at registration or in the protocol? 25) Did the review authors describe sources of financial or non-financial support for the review and the role of the funders or sponsors in the review? 26) Did the review authors declare any competing interests of review authors? 27) Did the review authors report which of the following are publicly available and where they can be found template data collection forms; data extracted from included studies; data used for all analyses; analytic code; any other materials used in the review? Scored as 0,1.	44	75%	Assaram et al. 2017 [18]
Newcastle Ottawa [16]	Cohort	Eight items: 1) Representativeness of the exposed cohort, 2) Selection of the non-exposed cohort, 3) Ascertainment of exposure, 4) Demonstration that outcome of interest was not present at the start of study, 5) Comparability of cohorts based on the design or analysis*, 6). Assessment of outcome, 7) Was follow-up long enough for outcomes to occur, 8) Adequacy of follow-up of cohorts Scoring was done by placing a point on each category. Scored as 0, 1, 2.	8	75-83%	Campbell et al. 2009 [19], Choi et al. 2007 [20]
SANRA [17]	Narrative Review	Six items: 1) Justification of the article’s importance to the readership, 2) Statement of concrete aims or formulation of questions, 3) Description of the literature search, 4) Referencing, 5) Scientific reason, and 6) Appropriate presentation of data. Scored as 0, 1, or 2.	12	75-92%	Alfano et al. 2019 [21], Bertoldi et al. 2017 [22], Bruggerman et al. 2009 [23], Campos et al. 2016 [24], Derek et al. 2008 [25], Kalim et al. 2008 [26], Kopp et al. 2003 [27], Naicker et al. 2015 [28], Reghine et al. 2008 [29], Roling et al. 2006 [30], Ross et al. 2014 [31], Swanepoel et al. 2018 [32], Weiner et al. 2003 [33]

Results

**Figure 1:**
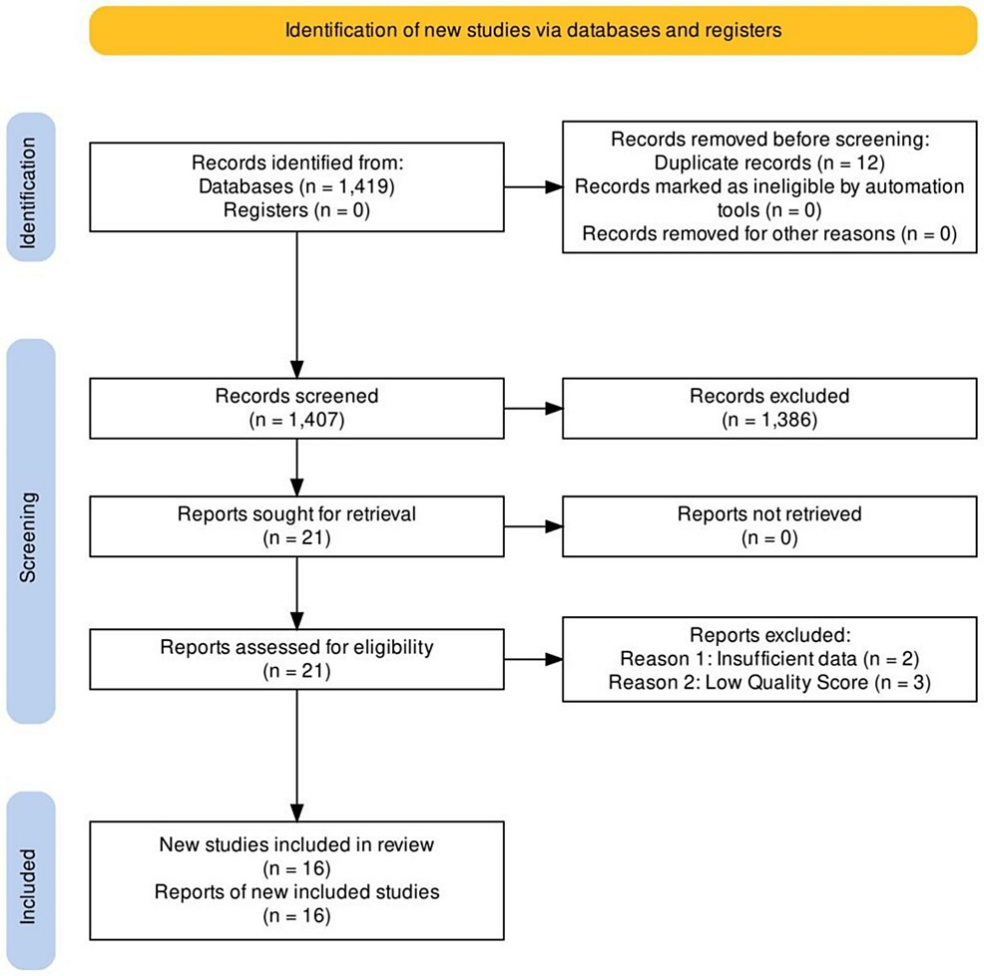
The article filtering processes are depicted in the PRISMA flow diagram. PRISMA: Preferred Reporting Items for Systematic Reviews and Meta-Analysis.

The comprehensive and exhaustive database searches of PUBMED, Google Scholar, and Cochrane revealed 1419 potentially related titles based on the study inclusion criteria for this systematic review. Removal of duplicates left 1407 articles, after which all remaining records were screened using the title and abstract. A secondary review was then carried out by reading full-text articles and the use of detailed inclusion and exclusion criteria to eliminate irrelevant articles not possessing the required data for this systematic review. This holistic evaluation yielded 21 articles on our research question, which were further subjected to the screening tools. After a quality appraisal, we eliminated five studies, and the final 16 articles were included in our systematic review. These articles comprised two cohort studies, one systematic review, and 13 narrative reviews. We created a PRISMA flowchart for study identification and filtering the articles as shown in Figure 1 [15]. Table 2 discusses the characteristics of the papers included in the study.

**Table 2:** Characteristics of papers included in the study CKD-EPI: Chronic Kidney Disease Epidemiology Collaboration; CKD: chronic kidney disease; HIV: human immunodeficiency virus; ART: antiretroviral therapy; CD4: cluster of differentiation 4, MDRD: modification of diet in renal disease; eGFR: estimated glomerular filtration rate; HIVAN: HIV-associated nephropathy; ESRD: end-stage renal disease; TDF: tenofovir disoproxil fumarate; RNA: ribosomal ribonucleic acid; mRNA: messenger ribosomal ribonucleic acid; cART: combined antiretroviral therapy; IDV: indinavir; TFV: tenofovir; VA: Veterans Health Administration; AKI: acute kidney injury; FSGS: focal segmental glomerulosclerosis; MALD: mapping by admixture linkage equilibrium; LTR: long terminal repeats; gag: group-specific antigen; pol: DNA polymerase; ESRD: end-stage kidney disease

Author	Year of Study	Type of Study	Methods	Limitations	Conclusion
Alfano et al. [21]	2019	Narrative Review	CKD-EPI equation used to calculate the estimated prevalence of CKD in the HIV population		Despite the advances brought about by the use of ART, kidney disease and the risk factors linked with it are still a leading cause of morbidity and mortality among HIV-infected individuals. Black race, older age, hypertension, diabetes, low CD4 T-cell count, and high viral load are risk factors for kidney in this population.
Assaram et al. [18]	2017	Systematic Review	Urine analysis, Estimated glomerular filtration rate, Renal biopsy, Risk factors for renal dysfunction, Clinical and histological responses to ART, A comparison of the CG and 4-v MDRD equations, the 2009 Chronic Kidney Disease Epidemiology Collaboration (CKD-EPI) formula, and the 4-v MDRD equation was performed to determine eGFR.	The majority of the studies that were examined took place in urban areas with easy access to medical facilities and labs. The exclusion of literature written in languages other than English was a necessary restriction on this study.	HIVAN was discovered to be the third most common cause of ESRD in African Americans between the ages of 20 and 64. Numerous studies have shown that the use of TDF reduces kidney function and advise monitoring renal function to avoid TDF neurotoxicity. To reduce nephrotoxicity and enhance outcomes in HIV-infected people, early diagnosis of proteinuria using reliable screening assays is crucial.
Bertoldi et al. [22]	2017	Narrative Review	Used flow cytometry to demonstrate the presence of the HIV receptor and co-receptors on the surface of immortalized podocytes. In silico HIV RNA hybridization tests on people revealed viral mRNA in tubular and renal epithelial cells that was both spliced and unspliced.		HIV-related kidney damage is caused by a number of different processes, including direct infection of renal cells, host defence against particular viral antigens, and long-term antiretroviral medication use. Clinicians should be aware of the potential kidney consequences of HIV infection and should think about routinely monitoring patients who exhibit risk indicators associated with CKD progression given the vast range of potential interactions between HIV, specific predisposing factors, and cART.
Bruggerman et al. [23]	2009	Narrative Review	Biopsy of the kidney taken from a patient with HIVAN. A purple stain is produced as a result of the in situ hybridization technique used to identify HIV-1 messenger RNA. The above-discussed CD4 promoter method was used to construct a number of transgenic models that purposefully eliminated certain HIV-1 genes.		HIV-associated renal disorders are the outcome of a complex interaction between immunological activation, genetic predisposition, and viral infection. Although data suggests that HIVAN is harmful for direct infection of renal parenchymal cells, local and systemic immune responses may cause or worsen renal illness.
Campbell et al. [19]	2009	Cohort Study	It was determined and reviewed how many patients at King's College and Brighton Hospitals in the UK have CKD. eGFR 60 mL/min for 3 months was used to identify CKD. To look at patterns in renal function before, during, and after exposure to indinavir (IDV) or tenofovir, longitudinal eGFR slopes were created (TFV).	Ascertainment and review of CKD cases among patients attending King's College and Brighton Hospitals, UK were carried out. CKD was defined as eGFR <60 mL/min for ≥3 months. Longitudinal eGFR slopes were produced to examine trends in renal function before, during, and after exposure to indinavir (IDV) or tenofovir (TFV).	The significance of metabolic and vascular disease to the burden of CKD in an aging HIV-infected cohort is highlighted by this study. Treatment with IDV or TFV was linked to a faster loss of renal function in patients who acquired CKD.
Campos et al. [24]	2016	Narrative Review	Glomerular filtration rate, Serum creatinine-based eGFR (CKD-EPI equation), Serum cystatin C-based eGFR (CKD-EPI equation)		In conclusion, HIV infection has been linked to kidney harm. This is because kidney cells can get directly infected, or because the immune system reacts to viral antigens or opportunistic agents. cART, treatment for other infections, and the concurrent existence of other chronic conditions linked to renal impairment all increase the risk of kidney harm
Choi et al. [20]	2007	Cohort Study	Worked with a group of patients who met the criteria for CKD stage 3 or above as defined by the National Kidney Foundation. Between October 1, 2000, and September 30, 2001, 2 352,584 veterans between the ages of 18 and 100 had at least one outpatient serum creatinine measurement at a Department of VA facility. 11,125 of these patients were omitted because they had already attained ESRD, which is defined as requiring continuous dialysis or a kidney transplant. Next, eGFR was determined based on age, sex, race, and serum creatinine using the condensed MDRD formula.		HIV infection has significant clinical repercussions for CKD patients. The rates of death, ESRD, and renal function decline varied significantly across HIV-infected individuals of the white and black races, despite the fact that HIV is a significant risk factor for mortality in both white and black CKD patients.
Derek et al. [25]	2008	Case report and review of literature	A case presentation with review of other related articles .		Regardless of the underlying cause of kidney illness, it is essential to detect it early by careful monitoring and to manage it effectively with a precise diagnosis, frequently with the assistance of a nephrologist
Kalim et al. [26]	2008	Narrative Review	AKI was described as a rise in serum creatinine lasting two days or more, with the magnitude of the increase depending on the creatinine level at the start of the condition.	There is a dearth of information on hyponatremia and other electrolyte abnormalities in the ART era, making direct comparisons challenging.	Despite reductions in morbidity and mortality during the ART era, AKI is still a frequent condition in HIV-infected patients. Optimizing immunological status, recognising and aggressively treating diabetes, chronic kidney disease, and hepatitis co-infection are all essential components of successful prophylaxis in addition to identifying patients at risk for AKI.
Kopp et al. [27]	2003	Narrative Review	Examining potential genes for polymorphisms connected to disease is one strategy. Another method identifies the causative gene or genes by a genome-wide scan that depends on linkage disequilibrium between DNA markers and the illness gene. Mapping by admixture linkage disequilibrium is a method for identifying disease genes in admixed populations that takes advantage of linkage disequilibrium between disease genes and marker genes (MALD).		A typical side effect of HIV-1 infection, HIV-associated FSGS responds regularly to anti-retroviral therapy. Direct viral infection of renal parenchymal cells and/or the toxicity of viral accessory proteins are two likely pathomechanisms. Patients of African heritage are particularly susceptible to HIV-associated FSGS. Although the genetic locus or loci causing this susceptibility are still unknown, MALD is an appealing tool to apply in the search.
Naicker et al. [28]	2015	Narrative Review	Renal Biopsy Urinalysis		The burden of the disease will be reduced by early detection of kidney disease through introduction of screening following the diagnosis of HIV infection and annual screening subsequently, as well as by providing individuals who need it with access to cART. In order to prevent this disease, which is fatal without treatment and is linked to prolonged survival with cART, programmes for HIV infection prevention are crucial.
Reghine et al. [29]	2020	Narrative Review	Serum creatinine was the preferred biomarker for determining the glomerular filtration rate. Using electronic microscopy during a renal biopsy to observe the kidney tubules		Despite the virus's known interaction with the host and the fact that HIVAN is a side effect of drugs, this is how it is currently understood. Additionally, it has been observed that concomitant conditions including diabetes mellitus and hypertension, which are not solely linked to the virus, are associated with an increase in renal injury.
Roling et al. [30]	2006	Narrative Review	Information from a recent study that examined the incidence and cause of 754 HIV patients in a prospective analysis	There are numerous processes that might contribute to renal pathology in HIV-infected individuals, which can result in a wide range of clinical diseases. HIVAN and thrombocytopenic purpura formation appear to be directly mediated by HIV. Other virus-related pathophysiological mechanisms include side effects such renal immune complex deposition and indirect viral impacts. Long-term survival likely adds to a rise in secondary kidney damage, such as hypertensive nephrosclerosis and diabetic glomerulopathy, as well as HAART-induced metabolic changes, diabetes, and hypertension.	
Ross et al. [31]	2015	Narrative Review	Rats and mice that express an HIV transgenic controlled by the endogenous viral LTR promoter but lacking the gag and pol genes experience kidney disease that phenocopies the clinical and histopathological signs of HIVAN. Renal Biopsy		In conclusion, persons with HIV are more likely to develop a range of acute and chronic renal problems. Pathomechanisms such as direct viral-mediated harm, host genetic variables, concomitant disorders, and exposure to nephrotoxic chemicals are responsible for this increased sensitivity to kidney disease. To better comprehend these influences and create more efficient preventative and therapeutic approaches for kidney disease in this vulnerable population, more study is required.
Swanepoel et al. [32]	2018	Narrative Review	Studies in the general population are extrapolated to create clinical recommendations for the prevention and management of CKD in HIV-positive people. Renal biopsy The glomerulus as seen through electron microscopy	These suggestions, which incorporate combined clinical experience, information from observational studies, and laboratory research, reflect the expert opinion of conference attendants in the absence of data from randomized controlled trials.	ART has boosted survival, but HIV-positive people still have a higher risk of renal damage.
Weiner et al. [33]	2003	Narrative Review	Renal biopsy, Electron microscopy		The prognosis for HIV-infected patients with renal impairment is still rather bad despite recent improvements in their management.

Discussion

Renal disease is a significant complication of HIV infection, with a prevalence of 2.4-17% [20]. These disease entities result directly from viral nephrotoxic effect, leading to the histologic features of HIV-associated nephropathy (focal collapsing glomerulosclerosis), thrombotic microangiopathy, and immune complex glomerulonephritis, or indirectly due to antiretroviral medications such as indinavir and tenofovir, which have a nephrotoxic effect [30]. These, in turn, cause acute or chronic kidney disease. Acute renal failure is often a characteristic finding in HIV-infected patients. It is usually seen in advanced stages of HIV infection (i.e., CD4 cell count of <200 cells/mm^3^ and HIV RNA level of >10,000 copies/mL), hepatitis C virus co-infection, and a history of antiretroviral treatment [30]. Furthermore, the cause of chronic renal failure in HIV-infected patients can be challenging to assess on clinical grounds alone and can usually only be determined by renal biopsy {30}. 

This finding was buttressed by Islam et al., who conducted a systematic review and meta-analysis showing that the pooled relative risk of kidney disease among PLHIV was 3.87 (95%CI: 2.18-6.85) [34]. Jacobson et al. also reported in their study that the relative risk of kidney disease among 542 HIV-infected men with the clinical finding of abnormal proteinuria, having chronic kidney disease (CKD) stages 3-5, to be 5.1 (95%CI: 2.9-8.9), compared to 661 HIV-seronegative men [35]. Estimates were adjusted by age, race, hypertension, and diabetes. Bah et al. carried out an observational study to determine the frequency of renal diseases in HIV patients, with 45 (41.7%) demonstrating renal diseases. Renal complications in these cases consisted of acute renal failure in 24 cases (53.33%), chronic renal failure in 13 cases (28.89%), nephrotic syndrome in four cases (8.89%), and interstitial nephritis in four cases (8.89%) [36]. However, renal biopsy was not done for histologic diagnosis in these cases.

An observational study by Campbell et al. involved 3439 patients, 81 (2.4%) of whom were diagnosed with CKD. Patients with CKD were found to be older, showed lower basal CD4+ T-cell counts, and in addition, had more regularly experienced an AIDS-defining illness. HIV-associated nephropathy (HIVAN) is predominantly found in Blacks, supported by Campbell et al.'s study, in which HIVAN was diagnosed in 16 (62%) of 26 Black patients and none of 55 White/other ethnicity patients [22]. In addition, increasing age and comorbidities usually seen in CKD patients in the general population, such as diabetes mellitus, atherosclerosis, and hypertension, are also increasingly linked with CKD in HIV seropositive patients. 

Pathogenesis of HIV Renal Disease

Gray areas remain in data concerning the pathogenesis of renal diseases associated with HIV infection. Current literature suggests that the host response to chronic HIV infection may include continuous antibody synthesis and atypical cell-mediated immune responses, which directly determine renal pathologic outcomes [33]. The release of proinflammatory cytokines by HIV-infected lymphocytes and renal cells may also play a crucial role in mediating kidney injury [33]

The mechanism of entry of HIV-1 into renal cells is still poorly understood. This is because while HIV entry into renal cells occurs in a CD4-dependent manner; these renal cells do not possess the classic receptors and co-receptors required for viral entry, such as CXCR-4 or CCR5 (co-receptors for gp120), unlike macrophages, lymphocytes or dendritic cells [21].

Cell-to-cell interaction involving helper T cells has often been implicated in HIV infection of renal epithelial cells. Many studies describe the virological synapses that permit HIV to pass from infected CD4+ T cells to renal epithelial cells in a contact-dependent manner. This infective transfer is duplex and bidirectional as the renal epithelial cells can directly infect CD4+ T cells [37]. In keeping with this hypothesis, viral transmission occur more effectively when cells are infected in the presence of T cells, compared to cell-free viral inoculum [20]. Studies also showed that heparan sulfate proteoglycans (HSP), syndecan 1, and agrin might aid in this transfer, as using HSP inhibitors remarkably reduced infection of renal tubular epithelial cells [38].

Renal Complications of HIV infection

Several renal affectations occur with HIV infection, such as HIVAN, HIV-associated thrombotic microangiopathy (TMA), and HIV-associated immune-mediated glomerulonephritis [39]. 

HIVAN: Studies have shown that HIVAN is one of the major causes of end-stage renal disease (ESRD) in HIV seropositive patients, often progressing to ESRD without combined antiretroviral therapy (cART). Its clinical presentation is characterized by azotemia and proteinuria without significant peripheral edema in patients with advanced HIV infection. At the same time, the kidneys have a characteristic enlargement with loss of corticomedullary differentiation on ultrasound scans [40]. A systematic review conducted by Assaram et al. involving 6595 participants concludes that HIVAN is the most common histological finding on renal biopsy. It is the third leading cause of ESRD among African Americans aged 20 to 64 years. This study also added that in 101 HIV-positive, ART-naive patients with renal failure, 57 presented with acute kidney injury (AKI), 21 with acute-on-chronic kidney disease, and 23 with CKD [19]. The histologic picture includes collapsing focal segmental glomerulosclerosis (FSGS), microcytic tubular dilation, fibrosis, and interstitial inflammation [40]. The collapsed glomerulus is often seen along with proliferation, hypertrophy, and hyperplasia of the superimposed glomerular epithelial cells (podocytes), which tend to fill the urinary space, giving rise to the classical histologic picture of podocyte effacement [18]. Glomerular collapse is defined as at least one glomerulus with the collapse of glomerular basement membranes accompanied by proliferated glomerular epithelial cells [32]. In addition, the podocytes may proliferate to form a pseudo-crescentic structure [27]. HIVAN occurs mainly in African Americans with apolipoprotein L1 (APOL1) risk alleles and is a leading cause of ESRD in African Americans [3]. Disease expression and renal complications, and subsequent kidney injury risk require two APOL1 risk alleles. The presence of high-risk genotypes in healthy populations points to the fact that this disease expression requires a "second hit," such as infections (e.g., HIV, viral hepatitis, and others), interferon, gene-gene interactions, illicit drug use, and other CKD risk factors [32]. It is also strongly linked with low CD4+ T-cell count and South African populations [18]. HIVAN affects 27% of seropositive individuals, and in the absence of highly active ART (HAART), it quickly progresses to ESRD [20]. A finding in HIVAN is also absent or minimal immune complex deposition [32]. Tubulointerstitial disease is also an important component of HIVAN and often appears out of proportion to glomerular disease, causing renal enlargement and a hyperechoic picture on ultrasonography [32].

Non-Collapsing FSGS: This is also known as FSGS not otherwise specified (NOS). Cases of non-collapsing FSGS usually have a less severe degree of podocyte effacement [32]. The main histologic finding is segmental sclerosis of capillary loops with matrix increase (with or without hyalinosis) within the glomerulus and adherence of capillary tuft to Bowman's capsule [18]. In addition, it possesses a different epidemiology as it is more commonly seen in Caucasians than in African Americans [18].

HIV-Associated Immune-Mediated Glomerulonephritis: Similar to non-collapsing FSGS, immune-mediated glomerulonephritis is more common in caucasian populations than African American populations. This collection of immune-mediated renal pathologies is also known as HIV immune complex kidney disease (HIVICK). Gernholtz et al. performed a retrospective study on 104 renal biopsies in Johannesburg, South Africa, and discovered that 20% of these biopsies were classified as HIVICK [41]. The limitation of this study is that it was a retrospective study, and the data was limited by selection bias and a small sample size. They possess similar characteristics with lupus nephritis, including immunologic, histologic, and ultrasonographic features such as immunoglobulin (IgG, IgA, and IgM) and complement (C3 and C1q) mesangial deposits; however, it occurs in patients without negative serological findings and no clinical evidence of systemic lupus erythematosus (SLE) [42]. Electron microscopy often reveals subendothelial, intramembranous, and mesangial electron-dense deposits [33]. A study of 60 biopsy specimens found that some form of immune complex-mediated glomerulonephritis was present in 37% of biopsy specimens. These histologic findings are classified into immune complex-mediated glomerulonephritis, IgA nephritis, mixed sclerotic/inflammatory disease, and lupus-like syndrome [30]. IgA nephritis's histopathology and immunologic picture encompass segmental or diffuse mesangial matrix expansion with sub-epithelial and peripheral intramembranous electron-dense deposits [33].

HIV-Associated TMA: This includes the clinical findings of hemolytic uremic syndrome (HUS) and thrombotic thrombocytopenic purpura (TTP). Clinical features include microangiopathic hemolytic anemia, microangiopathic hemolytic thrombocytopenia, reduced haptoglobin, and schistocytes seen in peripheral blood smear [29]. Although the pathogenesis of HIV-related TMA is unknown, preliminary studies show that HIV infection causes injury to renal endothelial cells, leading to platelet activation and deposition in the renal microvasculature [25]. Coexisting infections, cytotoxins, deficiencies of platelet aggregation inhibitors, and coagulation cascade abnormalities have all been implicated in the pathogenesis of TMAs. [33]. Clinical presentation is proteinuria, hematuria complicated by rapid onset renal failure and damage, and multi-system organ dysfunction [29]. However, the incidence and prevalence have reduced since the advent of ART.

Other Renal Diseases in the Setting of HIV Infection

Renal diseases such as diabetic nephropathy and arterionephrosclerosis may occur in PLWH due to multiple risk factors. Studies have shown that HIV infection is associated with a four-fold increased risk of type 2 diabetes mellitus (T2DM) and poor glycemic control compared to uninfected people [18]. Conversely, T2DM can increase the risk and severity of renal manifestations in PLWH [18]. In addition, non-HIV-related kidney disease is also on the rise due to the aging patient population as a result of the prevalence of ARTs, which improve life expectancy. The life expectancy of highly educated PLWHs treated chronically with combined ART has reached that of the uninfected counterpart [43].

Limitations

This review was limited to studies written in the English language, and studies published after 2003. In addition, grey literature and non-free full texts were also excluded from the study, thereby reducing the amount of available data. We did not specify the locations of the studies. Also, there was a dearth of information on in-depth randomized clinical trials, clinical trials, and systematic reviews to demonstrate relationships between HIV infection and different aspects of renal diseases.

## Conclusions

This was a critical analysis of the link between HIV and renal disease, discussing the pathogenesis of HIV in renal disease, the various implications of renal disease, and complication triggers. One study was a systematic scoping review, two were observational studies, and the rest were narrative reviews. We also discussed the increasing incidence of non-HIV-associated renal diseases in PLWH, such as diabetic and hypertensive nephropathy. This results from the increasing life expectancy in PLWHs with the increased use of ART. Hence, there is a need for more research in the future to elicit complications of HIV further as it remains a critical public health topic.
